# The effect of an adaptation to hypoxia on cardiac tolerance to ischemia/reperfusion

**DOI:** 10.7555/JBR.36.20220125

**Published:** 2022-10-28

**Authors:** Natalia V. Naryzhnaya, Leonid N. Maslov, Ivan A. Derkachev, Huijie Ma, Yi Zhang, N. Rajendra Prasad, Nirmal Singh, Feng Fu, Jianming Pei, Akpay Sarybaev, Akylbek Sydykov

**Affiliations:** 1 Laboratory of Experimental Cardiology, Cardiology Research Institute, Tomsk National Research Medical Center, Russian Academy of Science, Tomsk, Tomsk Region 634012, Russia; 2 Department of Physiology, Hebei Medical University, Shijiazhuang, Hebei 050017, China; 3 Department of Biochemistry and Biotechnology, Faculty of Science, Annamalai University, Annamalainagar, Tamilnadu 608002, India; 4 Department of Pharmaceutical Sciences and Drug Research, Punjabi University, Patiala, Punjabi 147002, India; 5 Department of Physiology and Pathophysiology, National Key Discipline of Cell Biology, School of Basic Medicine, the Fourth Military Medical University, Xi'an, Shaanxi 710032, China; 6 Department of Mountain and Sleep Medicine and Pulmonary Hypertension, National Center of Cardiology and Internal Medicine, Bishkek 720040, Kyrgyzstan; 7 Kyrgyz-Indian Mountain Biomedical Research Center, Bishkek 720033, Kyrgyzstan; 8 Department of Internal Medicine, Excellence Cluster Cardio-Pulmonary Institute (CPI), Member of the German Center for Lung Research (DZL), Justus Liebig University of Giessen, Giessen, Hessen 35392, Germany; 9 Department of Mountain and Sleep Medicine and Pulmonary Hypertension, National Center of Cardiology and Internal Medicine, Bishkek 720040, Kyrgyzstan

**Keywords:** heart, vessels, infarct size, arrhythmias, chronic hypoxia

## Abstract

The acute myocardial infarction (AMI) and sudden cardiac death (SCD), both associated with acute cardiac ischemia, are one of the leading causes of adult death in economically developed countries. The development of new approaches for the treatment and prevention of AMI and SCD remains the highest priority for medicine. A study on the cardiovascular effects of chronic hypoxia (CH) may contribute to the development of these methods. Chronic hypoxia exerts both positive and adverse effects. The positive effects are the infarct-reducing, vasoprotective, and antiarrhythmic effects, which can lead to the improvement of cardiac contractility in reperfusion. The adverse effects are pulmonary hypertension and right ventricular hypertrophy. This review presents a comprehensive overview of how CH enhances cardiac tolerance to ischemia/reperfusion. It is an in-depth analysis of the published data on the underlying mechanisms, which can lead to future development of the cardioprotective effect of CH. A better understanding of the CH-activated protective signaling pathways may contribute to new therapeutic approaches in an increase of cardiac tolerance to ischemia/reperfusion.

## Introduction

Despite intensive studies, the mechanism of chronic hypoxia (CH) effect on cardiovascular system and its significance in clinical practice are not entirely clear. In the mid-1960s, Poupa's group demonstrated for the first time that an adaptation to hypobaric hypoxia promoted an increase in tolerance of the isolated rat myocardium to anoxia^[1–4]^. In the USSR, Meerson's group confirmed these observations and reported that an intermittent regimen adaptation to CH markedly reduced the mortality in rats with a coronary artery occlusion (CAO) by five to six-fold and reduced infarct size by 35%^[5]^, and the incidence of ventricular arrhythmias and the duration of ventricular fibrillation were also decreased^[6]^.

However, the adaptation to hypoxia has resulted not only in positive but also negative effects. In 1971, it was reported that CH caused pulmonary hypertension and right ventricular (RV) hypertrophy^[7–9]^. It should also be mentioned that investigators often used different protocols for the adaptation to hypoxia. It was often unclear whether these investigators used chronic intermittent hypoxia (CIH) or chronic continuous hypoxia (CCH).

When comparing the duration of cardioprotective effects, it should be noted that the infarct-reducing effect of ischemic preconditioning persisted for only three days^[10–11]^ , while the infarct-limiting effect of CH persisted for up to five weeks^[12]^. The antiarrhythmic effect of CIH persisted for 14 days after hypoxic exposure^[13]^. Therefore, studies of the molecular mechanisms of the adaptation to hypoxia, both infarct-sparing and antiarrhythmic effects, may provide the stimulus for the creation of fundamentally new drugs for the prevention of acute myocardial infarction and sudden cardiac death.

## The antiarrhythmic effect of the adaptation to hypoxia

### The main manifestation of antiarrhythmic effect of chronic hypoxia

Meerson's group was the first to discover that CIH could prevent the appearance of ventricular arrhythmias in rats during CAO^[6]^. The adaptation to intermittent hypoxia led to a decrease in the incidence of ventricular extrasystoles in patients with neurogenic ventricular extrasystoles^[14–15]^. It was demonstrated that CIH enhanced cardiac tolerance to the arrhythmogenic impact of epinephrine^[16]^. It was also demonstrated that CIH could prevent the appearance of both ischemic and reperfusion ventricular arrhythmias in rats^[13]^. Continuous hypoxia over a 28-day-period also prevented the arrhythmogenic effect on CAO and reperfusion in rats^[13]^. The antiarrhythmic effect of CIH persisted for 14 days after hypoxic exposure and disappeared 21 days after CIH^[13]^. The antiarrhythmic effect of CIH was confirmed in the isolated perfused rat heart, which was subjected to low-flow ischemia and electrical pacing^[17]^. It was reported that metabolic syndrome, induced by a fructose-fed diet in rats, promoted an increase in the incidence of reperfusion ventricular arrhythmias; however, CIH could prevent these appearances in both the control and fructose-fed diet groups^[18]^. Action potential duration (APD) was significantly shortened in fructose-fed rats, but prolonged in CIH rats, compared with the control rats^[18]^. It was also reported that CIH decreased the incidence of ischemic and reperfusion arrhythmias in dogs^[19]^. In a study performed in the isolated perfused rat heart, it was shown that CCH prevented the occurrence of reperfusion ventricular arrhythmias^[20]^.

It should be noted that Morand *et al* have reported that CIH can increase the incidence of ventricular fibrillation in CAO (30 min) in rats^[21]^. In this case, the used adaptation method differed significantly from that of other investigators. Rats were exposed to 14 days of a short period CIH (30 s at 5% O_2_ and 30 s at 21% O_2_, 8 h per day)^[21]^. Prolongation of the time of hypoxia and reoxygenation into minutes and milder hypoxia (9.5%–10% O_2_ for 5 to 10 min/cycle, with intervening 4 min normoxia, 5 to 8 cycles/day for 20 days) induced the antiarrhythmic effect on CAO and reperfusion in rats^[22]^ and dogs^[23]^. Naryzhnaya's group subjected rats to 30 sessions of CIH in an altitude chamber: 6 h per day, 5 days a week (except for Saturday and Sunday) over 6 weeks^[24]^. Atmospheric pressure in a chamber was reduced stepwise to 625 mmHg (hypoxia day 1), 560 mmHg (day 2), 505 mmHg (day 3), 462 mm Hg (day 4), and 405 mmHg (day 5 and all other days of hypoxia)^[24]^. The pressure of 405 mmHg simulated an altitude of 5000 m above the sea level. Investigators demonstrated that this adaptation prevented the appearance of ventricular fibrillation in rats with CAO. A similar protocol of adaptation was used by other investigators who reported an antiarrhythmic effect of CIH^[6,13,17,25–26]^. Utilizing CCH (10% O_2_), Neckar *et al* observed a considerable reduction in all forms of arrhythmias after five days of hypoxia. However, in rats exposed to CCH for 15 and 30 days, arrhythmias did not differ from normoxic controls^[27]^. It should be noted that the Russian group could not demonstrate any antiarrhythmic effect of continuous hypoxia (12% O_2_ for three weeks) (unpublished observation). The aforementioned studies demonstrate that CIH may result in both the antiarrhythmic and arrhythmogenic effects dependent upon which the adaptation protocol was chosen.

### The mechanism of the antiarrhythmic and arrhythmogenic effects of adaptation to hypoxia

In 1998, it was documented that CIH elicited cardiac tolerance to the arrhythmogenic impact of epinephrine, which was mediated *via* the activation of opioid receptors (ORs)^[16]^. Naryzhnaya's group demonstrated that CIH enhanced cardiac tolerance to the arrhythmogenic impact of ischemia (10 min) and reperfusion (10 min) *via* activation of δ-OR in rats^[28]^. It was also demonstrated that µ-OR or κ-OR was not involved in the antiarrhythmic effect of CIH^[28]^. We hypothesized that the antiarrhythmic effect of the adaptation to hypoxia could occur as a consequence of the increased antioxidant protection of the heart. Studies showed that CIH and CCH increased superoxide dismutase (SOD) activity in the myocardium and reduced the malondialdehyde (MDA) level in the heart during ischemia/reperfusion (I/R)^[13]^, while chronic administration of the selective δ-OR antagonist naltrindole abolished the antiarrhythmic effect of CIH in dogs, and chronic administration of antioxidant N-acetylcysteine had the same effect^[19]^.

**Figure 1:**
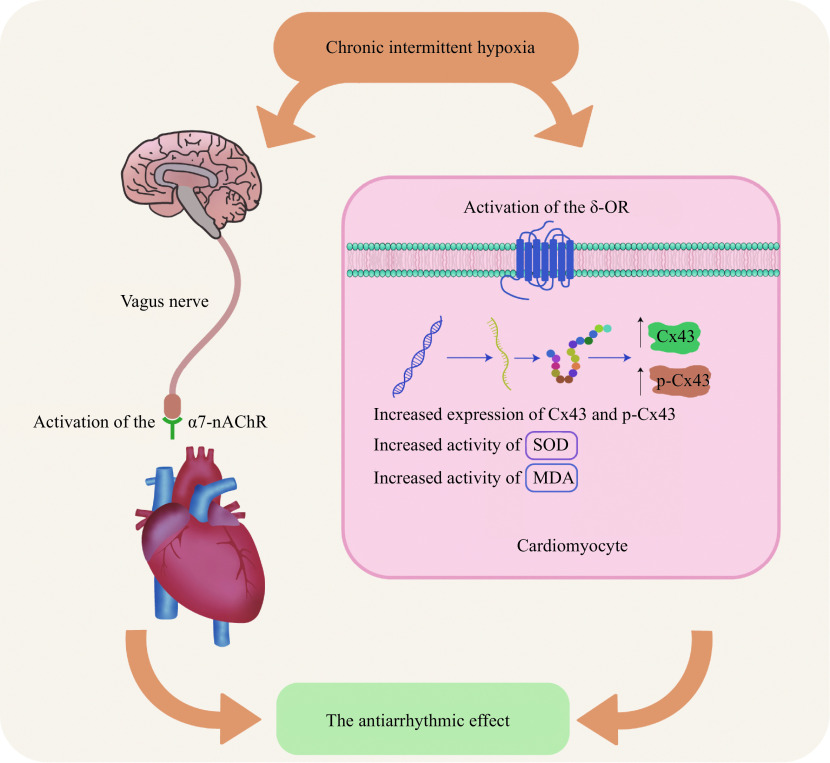
The possible mechanisms of the antiarrhythmic effect of chronic intermittent hypoxia.

It was demonstrated that the muscarinic receptor (mAChR) density was increased on the sarcolemma of cardiomyocytes during adaptation of rats to hypobaric hypoxia (a simulated altitude of 4250 m, 4 weeks)^[29]^, while non-selective muscarinic (M) receptor agonists oxotremorine and methacholine could prevent the appearance of ventricular fibrillation induced by CAO in dogs^[30]^. Recently, it was found that CH increased M2-receptor protein expression in rat atria^[31]^. Based on these studies, M-receptors are proposed to be involved in the antiarrhythmic effect of CH (***Fig. 1***).

Naryzhnaya's group documented that the nicotinic acetylcholine receptor (nAChR) antagonist hexamethonium abolished the antiarrhythmic effect of CIH in rats with I/R of the heart^[24]^. This investigation indicated that the autonomic nervous system was involved in the antiarrhythmic effect of CIH. We hypothesized that CIH increased vagus nerve activity and could contribute to the increased cardiac tolerance to the arrhythmogenic impact of I/R^[32–33]^. It has also been proposed that the antiarrhythmic effect of vagal stimulation is mediated *via* the M3-cholinergic receptor^[34]^. However, if the vagus nerve was involved in the antiarrhythmic effect of CIH, bradycardia should be expected. However, investigators observed a 13% increase in heart rate in mice after CIH^[35]^. Sherpas living at an altitude of 5000 m have an increase in heart rate and higher blood pressure, compared with people living at sea level^[36]^. Other investigators reported that CIH had no effect on heart rate in rats^[31,37]^. Therefore, it seems unlikely that M3-cholinergic receptor is involved in the antiarrhythmic effect of CH. However, the antiarrhythmic effect of CIH is likely associated with the activation of the α7 nicotinic acetylcholine receptor (α7-nAChR subtype), because stimulation of this receptor resulted in the antiarrhythmic effect^[38]^. One study has shown that CIH increases the expression of connexin-43 (Cx43) and phosphorylated Cx43 at Ser368, which facilitates cardiac conductivity^[25]^. CIH increased the action potential duration (APD_50_ and APD_90_) in cardiomyocytes by two-fold^[39]^. The arrhythmogenic effect of CIH appears to be associated with an increase in the plasma norepinephrine level^[21]^.

The aforementioned studies have demonstrated that endogenous δ-OR agonists and reactive oxygen species are involved in the antiarrhythmic effect of CIH. Enhancing antioxidant defenses of the heart also contributes to increasing the heart's tolerance to the arrhythmogenic impact of I/R. It is likely that the antiarrhythmic effect is mediated *via* the activation of both the vagus nerve and α7-nAChR. The increased expression of Cx43 and p-Cx43 may be involved in the antiarrhythmic effect of CIH. Prolongation of the action potential may also be involved in the antiarrhythmic effect of CIH.

## Cardiac tolerance to ischemia-reperfusion injury elicited by chronic hypoxia

### The regimens and main manifestations of the cardioprotective effect of chronic hypoxia

The first report on the infarct-reducing effect of CIH was published in 1973 by Meerson's group^[5]^. However, these investigators did not define the area at risk, which cast some doubt on the evidence obtained at that time. Furthermore, Naryzhnaya's group failed to detect the infarction-limiting effect of CIH utilizing Meerson's protocol^[40]^. In 2002, Kolar's group found that CIH in a hypobaric chamber (simulated the altitude of 5000 m for 8 h/day, 5 days/week, 7 weeks) resulted in a 15% decrease in the infarct size/area at risk (IS/AAR) ratio in rats^[41]^. The infarct-reducing effect was weak but significant. For this reason, it is likely that the Russian group was unable to detect it using a similar adaptation mode^[40]^. In comparison, CCH decreased the IS/AAR ratio by 60%^[42]^. The infarct-limiting effect of CCH was described for the first time by Kolar's group in 2003^[43]^. It was also documented that the infarct-sparing effect of CIH (a simulated altitude of 7000 m, 8 h/day, 35 exposures) persisted for five weeks in rats^[12]^. In this case, the IS/AAR ratio was decreased by 32%. The reason for such a long-lasting cardioprotective effect remains a mystery. The infarct-reducing effect of CIH was confirmed in later studies performed in rats^[22,40,44–48]^, dogs^[19,23]^, and mice^[49]^. The infarct-limiting effect of CСH was confirmed in later studies performed in rats^[27,42,50]^. It was demonstrated that a one-hour episode of normoxia eliminated the infarct-sparing effect of CCH^[27]^. It is possible that, for this reason, we could not detect the infarct size reduction using Meerson's protocol of CIH.

Interestingly, Moulin *et al* found that a short period of CIH mimicking sleeping apnea (21% to 5% FiO_2_, 60 s cycles, 8 h/day, 3 weeks) decreased cardiac tolerance to I/R in mice^[51]^. This CIH model was significantly different from the adaptation protocol used by Kolar's group^[45]^, Zhang's group^[13]^, and other investigators^[22,46,48–49]^. It is likely for this reason that an increase in the IS/AAR ratio after CIH in mice was found^[51]^. It was also demonstrated that intermittent short-term hypoxia/reoxygenation (repeated cycles of 10% O_2_ during 30 s per min, 4 h/day) reduced the infarct size in rats by about 40% after 14 days of adaptation^[52]^. This adaptation protocol was more moderate than that used by Moulin *et al*. It may be the reason why it had the infarct-reducing effect. The aforementioned studies have confirmed the reports that the infarct-sparing effect of CH depends on adaptation methods. Kasparova *et al*^[53]^ adapted rats to CCH (3 weeks), CIH (8 h/day, 3 weeks), and CIH (23 h/day 3 weeks and found that CCH resulted in a 30% decrease in infarct size; CIH 8 h/day decreased infarct size by 19%; CIH 23 h/day did not alter infarct size. Moreover, CCH and CIH 8 h/day increased the expression levels of mRNA transcript of HIF2α, MnSOD, Cu, and ZnSOD; CIH 23 h/day resulted in a reduction of the glutathione/oxidized glutathione (GSH/GSSG) ratio. Other adaptation methods did not alter the GSH/GSSG ratio^[53]^. These studies have demonstrated that CIH 23 h/day greatly enhances oxidative stress. It is likely that this oxidative stress prevents cardiac tolerance to I/R. Kolar's group reported that hypercapnia attenuated the infarct-sparing effect of CCH, and they hypothesized that CO_2_ inhibited reactive oxygen species (ROS) production^[43]^. It has now been documented that ROS, in moderate concentrations, may act as signaling molecules in the activation of the protective pathways in the CH hearts^[54]^.

The healing effect of CCH on postinfarction cardiac remodeling in rats with permanent CAO was also studied by Hrdlicka *et al*^[55]^, in which seven days post-myocardial infarction, rats were exposed to CCH (12% O_2_, 3 weeks). It was reported that CCH attenuated left ventricle (LV) dilation without any effect on the scar area.

The cardioprotective effect of the adaptation to hypoxia is formed not only at the level of the organism, but also at the level of the isolated heart. It was demonstrated that CIH enhanced tolerance of the isolated rat heart to I/R^[41,56–57]^, and CCH had the same effect^[58–60]^. Naryzhnaya's group found that CCH increased tolerance of isolated cardiomyocytes to hypoxia/reoxygenation^[61]^, while Kolar's group showed that CCH enhanced resistance of the isolated cardiomyocytes to the cytotoxic impact of NaCN^[62–63]^.

Ma's group studied the impact of metabolic syndrome on the development of the cardioprotective effect of CIH^[64]^. In this study, both female lean and obese Zucker rats were used, and rats were subjected to CAO and reperfusion; as a result, CIH improved the recovery of left ventricular contractile function, reduced infarct size, and enhanced antioxidant capacity in lean Zucker rats; however, CIH did not result in the cardioprotective effect in Zucker rats with metabolic syndrome.

The aforementioned studies have indicated that CIH and CCH increase tolerance of the heart to I/R both *in vivo* and *in vitro*. CCH enhances the tolerance of the isolated cardiomyocytes to hypoxia/reoxygenation and induces a more pronounced infarct-reducing effect than CIH. These indicate that the main molecular events, which provide the cardioprotective effect of the adaptation to hypoxia, develop at the level of cardiomyocytes. CCH attenuates postinfarction cardiac remodeling, while metabolic syndrome prevents the development of the cardioprotective effects of CIH.

### The effect of chronic hypoxia on apoptosis and autophagy of cardiomyocytes

In one study, rats were exposed to hypoxia at a simulated altitude of 5000 m in a hypobaric chamber for 6 h/day, lasting 42 days, then isolated rat hearts were subjected to global ischemia (30 min) and reperfusion (60 min)^[65]^. Apoptosis was assessed by a number of terminal deoxynucleotidyl transferase-mediated dUTP nick end labeling (TUNEL) cells. CIH resulted in approximately a 75% decrease in the number of TUNEL positive cells^[65]^. It was also demonstrated that CIH resulted in approximately a 30% decrease in the number of TUNEL positive cells in the border zone of the infarct area^[47]^. Wang and Si used short-term intermittent hypoxia in rats^[52]^, and they found that this exposure condition during 7 and 14 days had no effect on the number of TUNEL positive cells in the myocardium after I/R, but aggravated apoptosis occured in the ischemic and reperfused heart after 28 days of the adaptation. Apparently, short-term intermittent hypoxic exposures are not very effective in preventing the apoptosis of cardiomyocytes at I/R of the heart. CCH reduced the expression of pro-apoptotic protein Bax and elevated the expression of anti-apoptotic protein Bcl-1 in the myocardium of rats^[66]^. Furthermore, CIH induced by short-term hypoxia/reoxygenation contributed to the apoptosis of cardiomyocytes after I/R of the heart in mice^[51]^.

It has been shown that CIH (four weeks) stimulates autophagy of cardiomyocytes in rats^[67–68]^. In the study of Chang *et al*, mice were exposed to CIH with an oscillation of the O_2_ concentration between 4% and 20% every 30 min for one to four days in an incubator^[68]^. CIH elevated the autophagy marker LC3-Ⅱ level in the myocardium without I/R. According to some investigators, CCH (10% O_2_, 28 days) could result in autophagy of cardiomyocytes in mice with I/R^[69]^. These investigators hypothesized that autophagy protected cardiomyocytes from endoplasmic reticulum stress and I/R cardiac injury^[68–69]^. The reduction of endoplasmic reticulum stress was also suggested to occur in hypertensive rats exposed to CCH (12% O_2_, four weeks)^[70]^.

The aforementioned studies suggest a need for further investigation of the role of autophagy stimulation and apoptosis inhibition in the cardioprotective effect of adaptation to hypoxia. Additionally, the effect of CH on pyroptosis and necroptosis of cardiomyocytes in I/R injury of the heart has not yet been studied.

### The effect of chronic hypoxia on cardiac contractility during ischemia/reperfusion

The main cause of death in patients with acute myocardial infarction, which accounts for 50% of the cases, is cardiogenic shock^[71]^, since there currently are no drugs capable of effectively preventing the occurrence of cardiogenic shock. We propose that studies of the molecular mechanism(s) of the inotropic effect of CH may accelerate the development of such drugs.

In 1991, it was demonstrated that CIH improved the recovery of contractility of the isolated rat heart during reperfusion^[72]^, which was confirmed by Kolar's group^[41]^ and other investigators^[65]^. The recovery of cardiac function of the isolated rat heart after I/R was improved, and infarct size in CIH rats was reduced, compared with those in control rats^[57]^. Other investigators also observed the improvement of reperfusion cardiac contractility after CIH^[37]^. Naryzhnaya's group found that CCH improved the recovery of cardiac contractility function during reperfusion of the isolated rat heart^[58–60]^. Thus, both CIH and CCH improve the recovery of postischemic myocardial function.

## The mechanism of the myocardial ischemic tolerance elicited by chronic hypoxia

Kolar's group has reported that the cardioprotective effects of CIH and ischemic preconditioning are not additive^[41]^, indicating that the end-effector of cardioprotective effects of CIH and ischemic preconditioning may be identical; however, in this case, it is not clear why the cardioprotective effect of CH persists for a month and the cardioprotective effect of ischemic preconditioning disappears after three days.

### The role of hormones and humoral factors in the cardioprotective effect of chronic hypoxia

**Figure 2:**
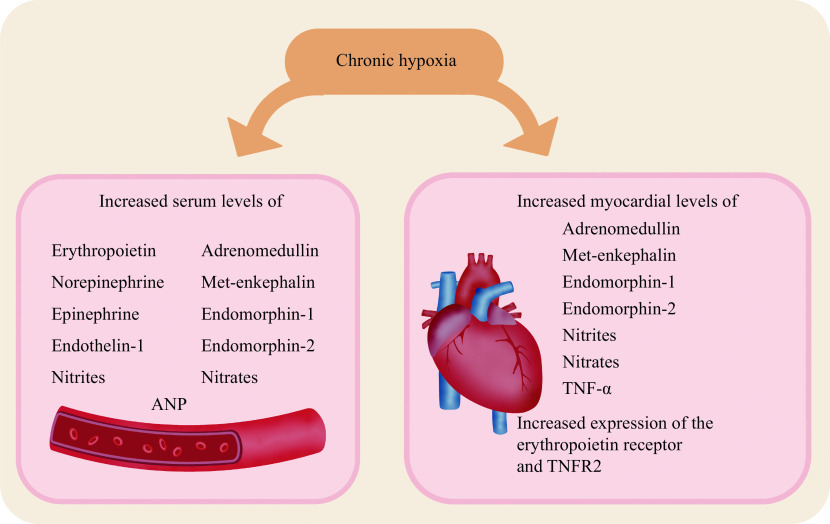
The effect of chronic hypoxia on the level of hormones and humoral factors in the blood and the expression of peptides in the myocardium.

One study showed that natriuretic peptides could increase cardiac tolerance to I/R^[73]^. Casserly et al found that CH (three weeks) resulted in RV hypertrophy, increased hypoxia-induced atrial natriuretic peptide (ANP) releasing from the isolated perfused rat heart, and increased cardiac tolerance to hypoxia^[74]^. The plasma ANP level was increased in hypoxaemic patients^[75]^ and in rats after hypoxia (seven days) (***Fig. 2***)^[76]^. These studies demonstrated that ANP could be involved in the cardioprotective effect of CH.

One investigation demonstrated that erythropoietin could prevent I/R cardiac injury^[77]^. Chronic high-altitude hypoxia induced an increase in serum erythropoietin level three-fold in human^[78]^. The plasma erythropoietin level was also increased in rats after two weeks of hypoxia^[79]^. The plasma concentration of erythropoietin in residents living at high altitudes was also increased^[80]^. Furthermore, CH increased the serum erythropoietin level and elevated myocardial erythropoietin receptor expression in rats^[81]^. Consequently, erythropoietin could be considered as a mediator of the infarct-limiting effect of CH.

Preliminary stimulation of β-adrenergic receptors (β-ARs) or α_1_-AR has been shown to increase cardiac tolerance to I/R^[82–83]^. Other studies showed that CIH increased the plasma concentration of norepinephrine and epinephrine in mice^[84]^ and norepinephrine in rats^[85]^. Therefore, it can be hypothesized that catecholamines may increase cardiac tolerance to I/R after CIH. Pretreatment with the β-AR antagonist trimepranol ameliorated the development of CIH-induced RV hypertrophy, and reduced the increased in RV pressure in rats^[86]^. Chronic administration of the β_1_-AR antagonist metoprolol during CIH abolished the infarct-reducing effect of CIH in dogs^[23]^, which is surprising since metoprolol is known to limit infarct size by itself^[87]^. It is logical to think that if endogenous catecholamines are involved in the infarct-sparing effect of CH, chronic administration of catecholamines can also enhance cardiac tolerance to I/R. However, it was found that chronic administration of the non-selective β-AR agonist isoproterenol [30 µg/(kg·day)] *via* an osmotic minipump decreased cardiac tolerance to I/R in mice^[88]^. It is likely that dose and administration methods play an important role in the beneficial and adverse effects of catecholamines.

In contrast, one study showed that the overexpression of α_1_-AR resulted in a cardioprotective effect in mice^[89]^. in which chronic hypobaric hypoxia (at a simulated altitude of 5500 m for 21 days) contributed to an increase in the α_1_-AR density and a decrease in the β-AR density in the LV of rats. At the same time, isoproterenol and forskolin-induced activation of adenylyl cyclase was decreased. However, pertussis toxin increased both basal and isoproterenol-induced adenylyl cyclase activity in hypoxia-adapted rats^[90]^.

Further investigation is required to fully detect the role of β-AR and α_1_-AR in the formation of cardiac tolerance in CH. It is likely that the use of AR antagonists, which does not alter the IS/AAR ratio, will accelerate the search for an answer to this question.

In 1996, one study demonstrated that endothelin-1 could mimic the cardioprotective effect of ischemic preconditioning *via* activation of the endothelin-A (ET_A_) receptor and stimulation of protein kinase C (PKC)^[91]^. In the same year, another study reported that endothelin-1 protected the isolated rat heart against I/R through activation of ET_A_ receptor, stimulation of PKC, and channel opening of mitochondrial ATP-sensitive K^+^ (mitoK_ATP_) ^[92]^. These results were later confirmed by Duda *et al*^[93]^. Recently, endogenous endothelin-1 and ET_A_ receptor were found to be involved in the cardioprotective effect of remote preconditioning in rats^[94]^. However, there is little evidence that the selective ET_A_ receptor antagonist BQ123 may increase cardiac tolerance to reperfusion in rabbits^[95]^. CH (10% O_2_ for four weeks) promoted an increase in the plasma endothelin-1 level in rats by 1.5-fold^[96]^. CIH (10% O_2_ for 21 days) resulted in a two-fold increase in the plasma endothelin-1 concentration^[97–98]^. In addition, adaptation to CIH led to an increase in ET_A_ expression in the heart tissue^[98]^. Consequently, endothelin-1 could be involved in the development of the adaptive cardiac tolerance to I/R. The chronic administration of endothelin receptor antagonists during adaptation could contribute to an answer to this question.

There is an experimental evidence suggesting that adrenomedullin can enhance cardiac tolerance to I/R^[99–100]^. CIH (10% O_2_, 8 h/day, 6 days/week) for four weeks resulted in a 2.3-fold increase in the plasma adrenomedullin concentration and a 2.7-fold increase in the adrenomedullin level in RV tissue of rats^[101]^. Other investigators also found that CH (30 days) caused an increase in the plasma adrenomedullin level in rats^[102]^. Therefore, there are reasons to believe that the adrenomedullin could be involved in the infarct-reducing effect of adaptation to hypoxia.

Chronic obstructive pulmonary disease (COPD) caused hypoxemia and promoted a decrease in the serum thyroid hormone levels in comparison with healthy controls^[103]^. It was reported that thyroid hormones stimulated oxygen demand by tissues^[104]^. Accordingly, a decreased thyroid hormone level contributed to a reduction in the tissue oxygen consumption. Based on these observations, it is logical to suggest that hypothyroidism could increase cardiac resistance to I/R, because hypothyroidism promoted a decrease in infarct size, reduced lactate dehydrogenase and creatine kinase release from the isolated rat heart subjected to I/R^[105]^.

Opioid peptides have also been involved in cardiac tolerance to I/R^[106]^. We found that CCH (12% O_2_ for 21 days) resulted in an increase in the met-enkephalin, endomorphin-1, and endomorphin-2 levels in plasma and in myocardial tissue^[50]^. Myocardial I/R enhanced these alterations; however, the β-endorphin level was not changed. The IS/AAR ratio was 60% less in the adapted rats than that in control animals. We have also reported that the cardioprotective effect of CCH is mediated *via* activation of µ- and δ_2_-ORs, while Κ-OR and δ_1_-OR are not involved in CCH-induced cardiac tolerance to I/R^[106]^. CCH improved reperfusion recovery of cardiac contractility and decreased reperfusion creation kinase release^[58–59]^. Both effects were mediated *via* µ- and δ_2_-OR stimulation. We also found that CCH increased tolerance of isolated rat cardiomyocytes to anoxia/reoxygenation^[61,107]^. The cytoprotective effect of CCH was mediated by µ- and δ_2_-OR activation. These results were confirmed by Pei's group, who demonstrated that CH (10% O_2_ for 4 weeks) had no effect on κ-OR expression in the rat heart^[108]^. The involvement of opioids in adaptation to hypoxia has also been confirmed by other investigators^[19,109–110]^.

It is now widely accepted that tumor necrosis factor-α (TNF-α) reduces cell survival in the unfavorable setting by inducing apoptosis and necroptosis^[111–112]^. The TNF-α antagonist alleviated cardiac I/R injury^[113]^. However, Kolar's group established that endogenous TNF-α could increase cardiac tolerance to I/R injury^[114–115]^. It has been shown that CCH increased the myocardial TNF-α level and TNF-α receptor 2 (TNFR2) expression^[114–115]^. Pretreatment with infliximab (monoclonal antibody against TNF-α, once a week) alleviated but did not abolish the infarct-reducing effect of CCH^[114]^. Infliximab decreased TNFR2 expression, the nuclear factor κB (NFκB) level, the inducible NO-synthase (iNOS level), and prevented CCH-induced myocardial oxidative stress^[114]^. These studies indicated that TNF-α could be involved in the cardioprotective effect of CCH.

Adenosine receptors may mediate the cardioprotective effect of ischemic preconditioning^[10]^. One of our studies indicates that these receptors are not involved in the infarct-limiting effect of CCH (unpublished data). The aforementioned studies indicate that opioid peptides, µ-, and δ_2_-OR can play an important role in the cardioprotective effect of CCH. It is likely that ANP, erythropoietin, catecholamines, endothelin-1, TNF-α, and adrenomedullin also play a role in the infarct-reducing effect of CH.

### The involvement of reactive oxygen species in the cardioprotective effect of chronic hypoxia

In the 1980s, it was generally accepted that free radicals existed solely to damage cells^[116]^. Now the attitude towards free radicals has been changed. It has been shown that they can act as signaling molecules that increase the heart's tolerance to ischemia and reperfusion during pre- and post-conditioning^[10,117]^. Consequently, there was reason to believe that they might be involved in the development of cardiac tolerance to I/R during CH.

Intermittent hypoxia can protect the isolated rat cardiomyocytes from H_2_O_2_-induced cell death^[118–119]^. For example, exposure to intermittent hypoxia (5% O_2_, 5% CO_2_, and 90% N_2_, 4 days, 30 min cycles) resulted in a significant decrease in ROS levels after their H_2_O_2_-induced increase^[119]^. In addition, an increase in the mRNA levels of Cu,Zn-superoxide dismutase (SOD) and Mn-SOD was observed. The mRNA levels of catalase and glutathione peroxidase (GP) did not change. Catalase and GP activities were significantly higher after hypoxia^[119]^. These data indicate that exposure to intermittent hypoxia leads to the stimulation of endogenous antioxidant defense. It has been confirmed that the chronic hypobaric intermittent hypoxia leads to an increase in the level of SOD in the myocardium and a decrease in the level of MDA^[64]^.

A human study showed that chronic hypobaric hypoxia increased the rate of ROS production by 38%^[120]^. Exposure to hypobaric CH (a simulated altitude of 4600 m, PIO_2_ 90 mm Hg, cycles 4 h) increased the formation of mitochondrial ROS and led to an increase in the activity of catalase, GP, and SOD^[121]^.

CIH (a simulated altitude of 7000 m, 8 h/day, 25 days) promoted a decrease in the IS/AAR ratio by about 50% in rats^[54,122]^. Chronic administration of the non-selective antioxidant N-acetylcysteine (NAC, 100 mg/kg/daily) reduced the infarct-sparing effect of CIH, but did not eliminate it completely and apparently because NAC itself reduced infarct size^[54,122]^. CIH may also cause an increase in the PKCδ level in cardiac tissue, and chronic administration of NAC abolished this effect of adaptation^[54]^. PKC plays an important role in cardiac tolerance to I/R^[11,123]^. Therefore, a decrease in the PKC activity can aggravate I/R cardiac injury. This infarct-reducing effect of PKC correlated with an increase in superoxide dismutase (*e.g.*, Mn-SOD) content in the myocardial tissue. NAC eliminated an increase in the Mn-SOD level in the heart, which could result in a decrease in cardiac resistance to I/R. CCH could induce myocardial oxidative stress (an increase in the 3-nitrotyrosine and MDA levels)^[114]^. The aforementioned studies indicate that ROS is involved in the development of CIH-induced cardiac tolerance to I/R. However, it is still uncertain which free radicals are involved in the cardioprotection and formation of cardiac tolerance to I/R.

### MicroRNAs and chronic hypoxia

MicroRNAs (miRNAs) constitute a large group of small non-coding RNAs with a length of 20 to 22 nucleotides, which are post-transcriptional regulators of gene expression in animals, plants, and protozoa^[124]^. miRNAs originate from regions of RNA transcripts and fold back into themselves to form short hairpins^[125]^. One miRNA can interact with another to inhibit the interaction of the mRNA with ribosomes, thus to accelerate enzymatic degradation of the mRNA^[124]^, which in turn contributes to a decrease in synthesis of protein molecules encoded by the mRNA. It has been proposed that miRNAs are involved in the regulation of the heart's tolerance to I/R^[126–127]^.

He *et al* reported that microRNA-138 (miR-138) expression was increased in the RV of those patients who have cyanotic congenital heart disease (CHD)^[128]^. They also reported that miR-138 prevented apoptosis of H9C2 cells under hypoxia by inhibiting JNK kinase^[128]^. CIH may cause a decrease in the miRNA-214 level in the rat myocardium^[129]^; however, it is unclear whether miRNA-214 is related to the heart's tolerance to I/R. Huang *et al* detected a reduced miR-184 level in samples of the RV of patients with cyanotic heart valve defects^[130]^. It was also demonstrated that a miR-184 inhibitor decreased H9C2 cell survival under hypoxia, but stimulated apoptosis of these cells due to the increased expression of caspase-3 and caspase-9^[130]^. These investigations provide indirect evidence that miR-184 provides cardiomyocyte tolerance to hypoxia. On the other hand, miRNA-199a-5p was markedly downregulated in the RV tissue of patients with cyanotic CHD and in human myocardial cells cultured in hypoxic conditions^[131]^, in which the miRNA-199a-5p-mimic enhanced apoptosis of cardiomyocytes under hypoxia and that the miRNA-199a-5p inhibitor alleviated apoptosis of cardiomyocytes under hypoxia^[131]^. These studies indicate that miRNA-199a-5p plays a negative role in regulating cardiomyocyte tolerance to hypoxia. One study showed that a higher level of miR-23b was detected in samples obtained by biopsy of the RV in patients with cyanotic CHD, compared with patients with acyanotic CHD; prolonged hypoxia induced an increase in miR-23b expression in H9C2 cells; miR-23b promoted hypoxia-induced apoptosis of H9C2 cells^[132]^, and investigators concluded that the overexpression of miR-23b could promote apoptosis of cardiomyocytes under hypoxia^[132]^. CIH of H9C2 cells caused cell hypertrophy and enhanced the expression of miR-31 and protein kinase Cε, and miR-31 was hypothesized to induce hypertrophy of cardiomyocytes^[133]^. Zhang *et al* reported that CIH induced an increase in the miR-21 level in atrial tissue^[134]^; however, it remains unclear whether miR-21 is related to an increase in cardiac resistance to I/R.

In summary, CH significantly changes miRNA expression in the myocardium. Under CH, the expression of miR-138, miR-23b, miR-31, and miR-21c is enhanced, but the miR-184 and miR-199a-5p levels are reduced. It has also been reported that miR-138 and miR-184 enhance cardiomyocyte tolerance to hypoxia, while miRNA-199a-5p and miR-23b enhance cardiomyocyte apoptosis under hypoxia.

### The involvement of NO-synthase in the cardioprotective effect of chronic hypoxia

NO-synthase (NOS) catalyzes nitric oxide (NO) synthesis, which plays a role of intracellular messenger and provides intracellular signaling^[135]^. NOS is involved in the ischemic pre- and post-conditioning phenomenon^[10,123,136]^; therefore, there is a reason to believe that CH causes an increase in NOS expression. In a number of studies, it was found that CH stimulated the expression of inducible NOS (iNOS)^[115,137–141]^ and endothelial NOS (eNOS) in the myocardium^[142–147]^. There is a report that CH increases the expression of mitochondrial NOS (mtNOS) in the heart^[148]^. The latter is neuronal NOS (nNOS) that is associated with the inner mitochondrial membrane^[149]^. La Padula *et al* reported that CH enhanced nNOS expression^[143]^. Other investigators report that CH does not affect nNOS expression^[150]^. However, there was a report that CH did not affect the eNOS level in the myocardium, but it reduced p-eNOS expression^[151]^. A decrease in eNOS expression in the myocardium after CH has been reported^[141]^. It has also been documented that hypoxia-inducible factor-1α (HIF-1α) acts as an inducer of iNOS protein synthesis in cardiomyocytes^[139,152]^. Thus, most investigators believe that CH increases both eNOS and iNOS expression in the myocardium.

It has been reported that CH causes an increase in NOS activity in the myocardium^[140,153]^. An increase in NOS activity was also demonstrated in the myocardium of children with cyanotic congenital heart defects^[137]^. Activation of NO production in CH has a generalized character, because it is observed not only in the myocardium but also in isolated mesenteric arteries^[154]^. A Russian group reported an increase in the level of NO metabolites, nitrites, and nitrates in serum and in the myocardium of rats after CCH^[42]^. An increase of nitrites and nitrates in the myocardium of rats that were subjected to CH was also noted by other investigators^[150]^. An increase in NO production provides an increase in the heart's tolerance to I/R, and the NOS inhibitor L-NAME eliminates the increased tolerance of the isolated heart to I/R in CH-exposed rabbits^[155]^. We found that L-NAME, a non-selective NOS inhibitor, eliminates the infarct-limiting effect of CCH^[42]^. The iNOS inhibitor S-methylisothiourea also blocks the infarct-reducing effect of CCH^[42]^, while the nNOS inhibitor 7-nitroindazole did not affect the cardioprotective effect of CCH^[42]^. The aforementioned studies demonstrate that CH results in an increase in NO production in the myocardium, and that the infarct-reducing effect of CH is associated with activation of iNOS.

### The role of kinases in the cardioprotective effect of chronic hypoxia

#### Protein kinase C

PKC is involved in the cardioprotective effect of ischemic preconditioning and postconditioning^[10,123]^. In infants with cyanotic heart defects and in newborn rabbits under hypoxia, translocation (activation) of PKCε to the particulate fraction of homogenates was found^[156]^. In rats that were exposed to hypoxia for a long time, there was an increase in the PKCδ, PKCε, and PKCζ levels^[157]^. However, the infarct-limiting effect of CIH was abolished after blockade of PKC with chelerythrine^[44]^. The selective PKCδ blocker, rottlerin, reduced but did not completely eliminate the infarct-sparing effect of CIH^[44]^. CIH induced translocation of PKCδ to mitochondria and the nucleus of cells. Naryzhnaya *et al* observed that rottlerin eliminated an adaptive increase in cardiomyocyte tolerance to anoxia/reoxygenation^[158]^. These studies indicate the important role of PKCδ in the cardioprotective effect of CH. CH has been shown to increase the level of PKC activator diacylglycerol in the myocardium^[159]^. At the same time, there is evidence that an increase in PKCδ activity could be the result of oxidative stress that is observed after exposure to CIH^[54]^. Thus, daily administration of the antioxidant N-acetylcysteine to rats eliminated the infarct-reducing effect of CIH and abolished the translocation of PKCδ to the particulate fraction from homogenates of the rat myocardium^[54]^. Indeed, the formation of nitrotyrosine, a marker of oxidative stress, was enhanced in the CIH myocardium, particularly in mitochondria^[160]^. The ability of ROS to activate PKC has been confirmed^[117]^, which provides a close correlation with results of the studies performed on rats that were adapted to CIH^[54]^. It should be noted that PKCδ is involved in the cardioprotective effect of CCH^[158]^, and the PKCδ level in the particulate fraction from homogenates of the rat myocardium is negatively correlated with infarct size after adaptation to hypoxia^[161]^. While CIH also increases the resistance of cardiomyocytes to anoxia/reoxygenation and reduces Ca^2+^ overload of cardiomyocytes after anoxia/reoxygenation^[57]^, Chelerythrine, a PKC inhibitor, eliminates these protective effects^[57]^.

There is evidence that PKCε is also involved in increasing the heart's tolerance to I/R after adaptation to continuous hypoxia^[63]^. In chronically hypoxic patients and animals, PKCε was activated along with p38 MAP kinase and JNK pathways^[162]^, which confers cardio-protection in the myocardium. Moreover, the activation of PKCε was accompanied by the inactivation of glycogen synthase kinase 3β (GSK3β) through phosphorylation, resulting in the increased accumulation of HIF-1α in the chronically hypoxic heart^[162]^. Because the activation of GSK3β aggravates I/R injury of the heart, its inhibition promotes an increase in cardiac tolerance to I/R^[163]^ The adaptation of rats to hypoxia led to an increase in tolerance of cardiomyocytes to 25-min metabolic inhibition, which was achieved by using NaCN and 2-deoxyglucose. It has been reported that CH contributes to the increased expression of PKCε. For example, the selective inhibitor, PKCε KP-1633, eliminated the cytoprotective effect of CH^[63]^. It is possible that other isoforms of PKC are involved in the cardioprotective effect of CH. It was also demonstrated that CIH increased PKCα expression and its phosphorylated (active) form in the myocardium of rats exposed to CIH^[164]^. CCH also promoted increased translocation of PKCβⅡ and PKCη into membranes of cardiomyocytes^[165]^. The aforementioned studies indicate the participation of at least two isoforms of PKC in the cardioprotective effect of CH, which was confirmed by using selective inhibitors: PKCδ and PKCε.

#### CaMK*Ⅱ* kinase

It was reported that the activation of Ca^2+^-calmodulin kinase Ⅱ (CaMKⅡ kinase) exacerbated I/R injury of the heart^[166–167]^. Consequently, there is a reason to believe that its activity decreased in the myocardium after CH. However, the expression of mRNAs encoding calmodulin, CaMKⅡγ, and CaMKⅡδ in the rat myocardium was enhanced after exposure to CH^[168]^. The increased CaMKⅡ expression after CH was also noted by other investigators^[169]^. A piece of evidence has been obtained that the increased CaMKⅡ expression may inhibit cardiomyocyte damage during the Ca^2+^ paradox^[170]^. It has been demonstrated that hypoxia (1% O_2_, 5% CO_2,_ and 94% N_2_, 12 h) leads to the activation of CaMKⅡδA and the phosphorylated type 2 ryanodine receptor (p-RyR2) in rat cardiomyocytes, while these effects were attenuated by knockdown of CaMKⅡδA^[171]^. In addition, CaMKⅡδA knockdown significantly reduced hypoxia-induced Ca^2+^ leakage from the sarcoplasmic reticulum of cardiomyocytes, and normalized hypoxia-induced downregulation of sarcoplasmic Ca^2+^-ATPase 2a (SERCA2a) expression in cardiomyocytes^[171]^. Thus, the inhibition of CaMKⅡδA can prevent Ca^2+^ release from sarcoplasmic reticulum by suppressing p-RyR2 and increasing SERCA2a expression. However, the available data are still insufficient to conclude that CaMKⅡ is able to provide a cardioprotective effect in adaptation to CH.

#### ERK and MEK kinases

The extracellular signal-regulated kinase (ERK) and mitogen-activated protein kinase kinase (MEK) play important roles in pre- and post-conditioning of the heart^[123]^. It has been hypothesized that these kinases may be involved in the infarct-sparing effect of CH. It has also been reported that CIH promotes an increase in ERK2 expression in the rat myocardium^[172]^. CIH resulted in an increase in the level of phosphorylated (active) ERK1/2 (p-ERK1/2) in the myocardium^[173]^. Inhibitors of MEK1/2 U0126 and PD-98059 eliminated the infarct-limiting effect of CIH^[173]^. Other investigators have noted an increase in the p-ERK1/2 level in the rat myocardium after CIH^[134,164]^. These studies indicate that both ERK1/2 and MEK1/2 are involved in the cardioprotective effect of CIH.

#### PI3 kinase and Akt kinase

Phosphatidylinositol-3-kinase (PI3K) and Akt kinase are also involved in pre- and post-conditioning of the heart^[123]^. It was demonstrated that CIH induced an increase in p-Akt expression in the LV of rats^[172]^, and the PI3K inhibitor LY294002 eliminated the infarct-limiting effect of CIH^[174]^. Furthermore, chronic moderate hypoxia induced an increase in the p-Akt level in H9c2 cardiomyoblasts^[175]^. In a study, researchers subjected the isolated perfused rat hearts to I/R, and found that the infarct-reducing effect of CIH was associated with PI3K activation^[173]^. Another study also documented that CCH increased the p-Akt level in the rat myocardium^[66]^. In contrast, CIH was found to induce a decrease in the PI3K and p-Akt levels in the rat myocardium and a decrease in p-PI3K expression^[134,176]^. A Russian group also failed to confirm the involvement of PI3K in the infarct-sparing effect of CCH^[42]^, and the PI3K blockade by wortmannin did not affect the increased tolerance of the isolated cardiomyocytes to anoxia/reoxygenation in rats that were adapted for CCH^[158]^. Thus, the role of PI3K and Akt in the cardioprotective effect of CH is controversial and remains to be clarified.

#### p38 kinase

The p38 kinase is also involved in pre- and post-conditioning of the heart^[123,177]^. CIH resulted in a decrease in the p38 and p-p38 kinase levels in the myocardium^[178]^, but CCH did not have a similar effect^[178]^. The p-p38 level in the RV decreased after CIH but increased in the LV, while the total p38 level remained unchanged^[172]^. An increase in the p-p38 level in the myocardium of the LV after CIH was observed in a later study by investigators of the same team^[164]^. Another study showed that the p-p38 level was increased in the myocardium of infants with cyanotic heart defects, but not in patients with acyanotic heart defects^[146]^. It was reported that the blocking p38 with SB203580 eliminated the cardioprotective effect of CCH^[156]^. In summary, the data on a role of p38 kinase in the cardioprotective effect of CIH is still controversial.

#### JNK

It is generally accepted that c-Jun N-terminal kinase (JNK) plays a negative role in the regulation of the heart's tolerance to I/R^[179]^. However, there is evidence that this enzyme is involved in the cardioprotective effect of remote preconditioning^[180]^. For example, curcumin, a JNK inhibitor and antioxidant^[181]^, eliminated the cardioprotective effect of CCH in rabbits^[156]^. However, an increase in the p-JNK level in the LV of rats adapted to CIH could not be detected^[178]^. Other investigators were also not able to detect changes in total JNK and p-JNK levels in the right and left ventricles as well as the isolated cardiomyocytes of rats adapted to CIH^[172,182]^. The presence of H9C2 cardiomyoblasts in a medium containing 1% O_2_ for 72 h contributed to an increase in the p-JNK level^[183]^. Furthermore, CIH led to an increase in the p-JNK/JNK ratio in the myocardium^[184]^. These studies indicate that CH may lead to an increase in the active p-JNK level in the myocardium. However, there is only one report indicating the involvement of this kinase in the cardioprotective effect of adaptation^[156]^. Therefore, the role of JNK in the protective effect of CIH remains to be investigated.

#### mTOR

There is evidence that rapamycin, an inhibitor of mTOR (mammalian target of rapamycin), eliminates the cardioprotective effect of ischemic postconditioning^[185]^. Therefore, there are reasons to suggest that mTOR may be involved in the cardioprotective effect of CH. A study performed with the isolated cardiomyocytes demonstrated that the *mTOR* mRNA and p-mTOR protein expression decreased after 48 h of hypoxia^[186]^. Another study has shown that CIH increases p-mTOR expression in rat cardiomyocytes^[187]^. Rapamycin is reported to abolish the cardioprotective effect of hypoxic preconditioning in cardiomyocytes *via* mTOR inhibition^[188]^. Thus, according to the available data, it is difficult to clarify the role of mTOR in the infarct-limiting effect of CH.

#### Protein kinase G

PKG (cGMP-dependent protein kinase G) is involved in the cardioprotective effect of pre- and post-conditioning^[123]^. Chronic hypobaric hypoxia caused an increase in the level of cGMP, a PKG activator, in the myocardium^[169]^ and the PKG upregulation in hypobaric CIH (a simulated altitude of 7000 m, 5 weeks)^[25]^. It remains unclear if CH contributes to an increase in the PKG activity, or what is the role of this kinase in the CH initiated infarct-limiting effect.

#### AMPK

ATP-activated protein kinase (AMPK) also plays an important role in pre- and post-conditioning phenomena^[123,177]^. It was reported that CIH (4 weeks) increased the p-AMPK level in rat cardiomyocytes^[67,189]^, while CCH upregulated the pAMPK/AMPK ratio during a brief ischemia protocol^[66]^. An increase in AMPK activity has been confirmed in the myocardium of infants with cyanotic heart disease, whose activity was also increased in H9c2 cells in CH (94% N_2_, 5% CO_2_, 1% O_2_, 48 h)^[190]^. In addition, AMPK activation in H9c2 cells stimulated mitophagy, which was eliminated by the inhibition of AMPK^[190]^. These data suggest that AMPK activation in CH improves mitochondrial quality and may play an important role in the cardioprotective effect of CH. Consequently, it may be hypothesized that AMPK is involved in the cardioprotective effect of CH.

#### GSK3β

It was demonstrated that phosphorylation of glycogen synthase kinase 3β (GSK3β) contributed to the inactivation of this kinase and increased cardiac resistance to I/R^[191]^, but CH did not affect the p-GSK3β level in the myocardium of mice^[192]^. Therefore, it has not yet been confirmed whether GSK3β plays a role in the infarct-limiting effect of CH.

#### Hexokinase

It was reported that the binding of hexokinase 2 (HK-2) to mitochondria prevented the apoptosis of cardiomyocytes^[123]^, and CIH results in the translocation of hexokinase to mitochondria^[193]^. An increase in the expression of HK-1 and HK-2 in the myocardium and their translocation to mitochondria were also reported^[193]^. CCH also increased the expression of HK-1 and HK-2 in the myocardium, and enhanced association of HK-2 with mitochondria^[66,70]^. Similar effects of CCH were detected in SHR rats^[70]^.

### The role of K_ATP_ channels and BK_Ca_ channel in the cardioprotective effect of chronic hypoxia

There are two main types of ATP-sensitive K^+^ (K_ATP_) channels: the sarcolemmal K_ATP_ (sarcK_ATP_) channel and the mitochondrial K_ATP_ (mitoK_ATP_) channel^[194–196]^. Both the K_ATP_ channel subtypes are involved in the cardioprotective effect of the ischemic pre- and post-conditioning^[136,194,196]^. Activators of K_ATP_ channels increased cardiac resistance to I/R^[195]^. Therefore, there was a reason to believe that K_ATP_ channels could be involved in the cardioprotective effect of CH.

In 1997, it was reported that CH caused a shortening of the action potential in the Purkinje fibers of the rabbit heart as a result of K_ATP_ channel opening^[197]^. The activation of the sarcK_ATP_ channel in response to CH was confirmed in a study, which was performed in isolated cardiomyocytes from mice with dominant-negative suppression of Kir6.2/SUR2A^[198]^. One study has shown that CIH increases resistance of the isolated rat heart to damage, which is caused by the Ca^2+^ paradox due to mitoK_ATP_ channel opening^[170]^. Crawford *et al* detected that the long-term moderate hypoxia of H9c2 cardiomyoblasts led to an increase in the resistance of these cells to hypoxia/reoxygenation^[199]^. In this study, HMR 1098, the selective inhibitor of sarcK_ATP_ channel, eliminated the cytoprotective effect of moderate hypoxia. Mild hypoxia contributed to an increase in the expression of SUR2A, which was a regulatory subunit of the K_ATP_ channel, but not a Kir6.2, a subunit forming the K_ATP_ channel pore. Overexpression of HIF-1α did not affect the SUR2A level^[199]^, which indicates that HIF-1α is not involved in the transcription of SUR2A mRNA. The mTOR inhibitor rapamycin did not affect the expression of SUR2A under moderate hypoxia. LY 294002, a PI3K inhibitor, and PD 184352, a MEK inhibitor, eliminated an increase in the SUR2A level caused by hypoxia. These studies indicate that SUR2A expression is activated with the involvement of PI3K and MEK in the setting of hypoxia. The non-selective K_ATP_ channel inhibitor glibenclamide eliminated increased resistance of the isolated heart of the adapted rabbits to hypoxia^[155]^. MCC-134, which is a blocker of the mitoK_ATP_ channel and an "opener" of the sarcK_ATP_ channel, eliminated the infarct-limiting and antiarrhythmic effects of CIH (a simulated altitude of 7000 m, 8 h/day, 7 weeks)^[45]^. These studies indicate an important role of the mitoK_ATP_ channel in the cardioprotective effect of CH. An important role of K_ATP_ channels in the infarct-limiting and antiarrhythmic effects of CH was confirmed in other studies^[40,200–201]^. Studies showed that glibenclamide and 5-hydroxydecanoate, the two selective mitoK_ATP_ channel inhibitors, eliminated the infarct-reducing effect of CCH (12% O_2_, 3 weeks)^[42,202]^, but the sarcK_ATP_ channel blocker (HMR 1098) had no effect^[42]^. It should be noted that Forkel *et al* using glibenclamide could not confirm the involvement of K_ATP_ channels in the increased tolerance of the RV to I/R in rats exposed to CH (10.5% O_2_, 2 weeks)^[145]^.

It has been documented that the mitochondrial big-conductance Ca^2+^-activated K^+^ (BK_Ca_) channel opening increases tolerance of cardiomyocytes to hypoxia/reoxygenation^[203]^. Borchert *et al* reported that the BK_Ca_ channel was involved in an increase in tolerance of the isolated cardiomyocytes to the metabolic inhibition and reenergization^[62]^. It may be proposed that the BK_Ca_ channel is involved in the cardioprotective effect of CH.

Thus, most studies indicate that the infarct-reducing effect of CH is associated with the mitoK_ATP_ channel activation. However, the reports, which indicate that sarcK_ATP_ and BK_Ca_ channels may provide an increase in CH-induced cardiac tolerance to I/R, cannot be excluded.

### The involvement of HIF-1α and nuclear factor-κB in the cardioprotective effect of chronic hypoxia

It has been reported that HIF-1α plays a key role in the development of adaptation to hypoxia^[204]^. HIF-1α may play an important role in the formation of the cardioprotective effect of CH. However, it is difficult to prove this, because the blockade of HIF-1α expression in the setting of hypoxia will inevitably lead to death of animals. The reason for an increase in the HIF-1α level in cells is not only due to hypoxia, but also to an increase in ROS production, because ROS inhibits prolyl hydroxylase that catalyzes rapid degradation of HIF-1α^[117]^. An increase in ROS production was observed in CH^[54,122]^. Investigators documented that the enhanced HIF-1α expression was associated with an increase in cardiac tolerance to I/R^[205–207]^. It was reported that CCH (10% O_2_ for 28 days) resulted in an increase in the expression of HIF-1α in myocardial tissue of mice^[69]^. However, CCH had no effect on the HIF-2α level in the murine heart^[69]^. CIH (a 6 h hypoxia per day for 28 days) resulted in an increase in HIF-1α expression in the heart of rats^[189]^. These results were confirmed by other investigators^[208]^. There was evidence that some effects of HIF-1α could develop rapidly during 1 h^[205]^. Therefore, it may be hypothesized that acute administration of HIF-1α inhibitors may abolish the CH-induced cardioprotection. HIF-1α is apparently not involved in CH-induced RV hypertrophy. It was found that pretreatment with the prolyl hydroxylase inhibitor dimethyloxalylglycine enhanced a CH-induced increase in the HIF-1α level in myocardial tissue, and attenuated RV hypertrophy and myocardial fibrosis, consequently, HIF-1α did not trigger RV hypertrophy in CH^[81]^.

NFκB is involved in both physiologic processes and diseases^[209]^. It regulates inflammation, immune function, differentiation, apoptosis, and cell survival^[209]^. NFκB was also involved in the infarct-reducing effect of ischemic preconditioning^[210]^. Exposing to CCH (12% O_2_ for 3 weeks) increased the NFκB level in myocardial tissue of rats by 70%^[114–115]^. However, the role of NFκB in the infarct-reducing effect of CCH remains to be clarified.

How HIF-1α and NFκB trigger an increase in the cardiac tolerance to I/R? It may seem strange, but the molecular mechanism of the HIF-1α-induced cardiac tolerance to I/R is practically not studied. There is an indirect evidence that HIF-1α may be involved in the activation of Akt, ERK and the inhibition of GSK-3β in cardiomyocytes and H9c2 cells^[70,211–213]^. The activation of HIF-1α could promote the mitochondrial permeability transition (MPT) pore closing in H9c2 cells^[212]^, and HIF-1α contributed to an increase in the expression of vascular endothelial growth factor in the rat heart^[213]^. It was shown that HIF-1α promoted antioxidant activity in the ischemic rat heart^[214]^. These events may promote an enhancement of the cardiac tolerance to I/R. It was reported that the activation of NFκB promoted an increase in the cardiac tolerance to I/R^[215–219]^. NFκB are involved in the delayed cardioprotective effect of volatile anesthetics, carbon monoxide, late ischemic preconditioning and a high-fat diet^[215–219]^. It was suggested that the cardioprotective effect of NFκB was mediated *via* an increase in the expression of cardioprotective proteins HSP70 family^[215–216]^. There is an indirect evidence that NFκB may trigger the expression of cardioprotective proteins, which are heme oxygenase-1 HO-1, cyclooxygenase-2, and superoxide dismutase as well as antiapoptotic proteins^[217]^. These data indicate that HIF-1α and NFκB may be involved in the cardioprotective effect of CH.

### Are mitochondria the end-effector of the cardioprotective effect of chronic hypoxia?

Mitochondria are the important source of ATP in cardiomyocytes; therefore, cardiac contractility in the setting of I/R depends on mitochondrial resistance to hypoxia/reoxygenation. At the same time, MPT pore opening induces cell apoptosis, a process by which mitochondria can induce cardiomyocyte death^[220]^.

CCH (12% O_2_ for 3 weeks) increased the isolated heart's tolerance to global ischemia (45 min) and reperfusion (30 min)^[58]^. The adaptation to hypoxia decreased creatine kinase release in coronary effluent during the reperfusion, and improved the reperfusion recovery of contractile function, mitochondrial respiration in state 3 as well as calcium retention capacity (CRC) compared to the normoxic group^[58]^. CRC characterizes MPT pore resistance to Ca^2+^-overload. Therefore, increased CRC may indirectly indicate an increased resistance of cardiomyocytes to apoptosis. The increased respiration rate in state 3 may be indicative of an improvement in ATP synthesis. Indeed, it was found that CCH promoted an increase in the myocardial ATP level during reperfusion compared to the normoxic group^[59]^. The non-selective OR antagonist naloxone (300 nmol/L) abolished the cardioprotective effect of CCH and the CCH-induced resistance of mitochondria to cardiac I/R^[58–59]^. The selective µ-OR antagonist CTAP and δ_2_-OR antagonist naltriben eliminated these protective effects of CCH, while the selective δ_1_-OR antagonist BNTX and κ-OR antagonist nor-binaltorphimine had no effect on the protective effects of CCH^[59]^.

The aforementioned studies indicate that CCH improves resistance of the heart and mitochondria to I/R. Opioid receptors (µ and δ_2_) play an important role in CCH-induced tolerance of mitochondria to I/R. It is possible that mitochondria may be the end-effector of the cardioprotective effect of CH.

In our opinion, mitochondria play a key role in the cardioprotective effect of CH, because they synthesize ATP, without which the life and normal functioning of the cell is impossible. The mitoK_ATP_ channel opening prevents cardiomyocyte death in the setting of hypoxia/reoxygenation^[221–222]^. The MPT pore closing prevents apoptotic death of cardiomyocytes in I/R of the heart, and increases cardiac tolerance to I/R^[220,223]^. It was found that the K_ATP_ channel openers reduced ROS production by mitochondria, and prevented H/R-induced injury of cardiomyocytes^[224–226]^. However, the prevailing evidence is that the K_ATP_ channel opening promotes an increase in ROS production^[227–231]^. The K_ATP_ channel opening also promotes activation of PKC^[232–234]^, and this enzyme plays a key role in cardioprotection^[163]^; thereby the activation of PKC after the K_ATP_ channel opening promotes an increase in cardiac tolerance to I/R. The K_ATP_ channel opener and a NO donor nicorandil (100 µmol/L) prevented MPT pore opening at oxidative stress in the isolated rat cardiomyocytes^[235]^. Many compounds, which increase cardiac tolerance to I/R, simultaneously opened K_ATP_ channels and closed MPT pore^[236–238]^. Maslov's group found that the infarct-reducing effect of opioid peptide deltorphin Ⅱ was mediated through the sarcK_ATP_ channel, and prevented MPT pore opening (unpublished data). We hypothesize that the K_ATP_ channel opening leads to MPT pore closure. However, the molecular mechanism interaction of K_ATP_ channels and MPT pore is unstudied yet. It is possible that mitochondria may be the end-effector of the cardioprotective effect of CH.

## The vasoprotective effect of chronic hypoxia

In one study, rats were exposed to CIH (9.5% to 10% O_2_ for 5 to 10 min/cycle, with intervening 4 min normoxia, 5 to 8 cycles/day for 20 days)^[22]^, and the isolated rat heart underwent global ischemia (15 min) and reperfusion (10 min). Endothelial function was then evaluated from the relaxation to acetylcholine of norepinephrine-precontracted aortic rings, and from an increase in coronary flow produced by acetylcholine in the isolated hearts. Endothelial dysfunction in the aorta was marked after cardiac I/R in the non-adapted rats but was not significant in the adapted rats. Acetylcholine induced an increase in coronary flow without I/R, which was much weaker if the heart was subjected to I/R. CIH prevented coronary flow reperfusion fall, and inhibited a reperfusion decline in an acetylcholine-induced increase in coronary flow^[22]^. One study indicated that CIH could increase coronary flow of the isolated rat heart without I/R^[41]^ and CIH also improved postischemic recovery of coronary flow in rats^[57]^. It was also documented that CIH increased capillary density in the heart^[47]^ and collateral blood flow in the human heart^[239]^. However, there is no evidence that the adaptation to hypoxia enhances collateral blood flow in the myocardium of animals. Consequently, CIH exhibits the vasoprotective effect during I/R of the heart but enhances angiogenesis in the heart. Whether CCH has a vasoprotective effect in cardiac I/R is unknown.

## Perspectives for the clinical use of the adaptation to hypoxia

It was known that CIH (490 mm Hg) induced a decrease in heart rate, increased left ventricular ejection fraction, and decreased both systolic and diastolic pressures in patients with the coronary artery disease (CAD)^[15]^. Exposing patients with CAD to CIH (a simulated altitude of 3500 m, for 3 h daily, for 22 days) resulted in a decrease in the serum levels of total cholesterol, triglycerides, and low-density lipoprotein; in contrast, the high-density lipoprotein concentration was increased^[240]^. Surprisingly, these changes in lipid concentrations persisted for three months after returning to normoxic conditions^[240]^. In 2005, one study of patients with and without COPD showed that the mean cardiac collateral score was 2.15 ± 2.03 in the COPD group and 1.32 ± 1.54 in the control group (*P* = 0.002)^[239]^. These results indicate that CH can improve the coronary blood flow in the heart, which can be beneficial for patients with CAD. Patients with CAD underwent 14 sessions of CIH (a simulated altitude of 4200 m, 4 h/session)^[241]^, which improved myocardial perfusion in patients with severe CAD. Patients with CAD were included in a clinical study, in which the duration of one procedure was 45 to 50 min, each hypoxic period (12% O_2_) was on average 4 to 6 min, while the hyperoxic period (35% O_2_) was 1 to 2 min, and these patients were exposed to 15 sessions over three weeks^[242]^. Exercise tolerance after the course of CIH increased and remained significantly elevated during the subsequent month^[242]^. When patients with CAD were subjected to hypoxia (10%–12% O_2_) and to hyperoxia (30%–35% O_2_), 3 sessions a week, 5 to 7 hypoxic periods lasting 4 to 6 min, with 3-min hyperoxic recovery intervals^[243]^, CIH increased exercise capacity, reduced both systolic and diastolic blood pressures, and enhanced LV ejection fraction. These studies indicate that CIH may have potential clinical utility for treatment of CAD, but a treatment protocol must be very carefully designed.

## Conclusions

It was demonstrated that CIH could prevent the arrhythmogenic impact of I/R on the heart. The antiarrhythmic or arrhythmogenic effect of CIH depends on protocols of the adaptation to hypoxia. The antiarrhythmic effect is mediated *via* the activation of n. vagus, M3-cholinergic receptors or α7-nAChR, and the stimulation of the δ-OR by endogenous opioid peptides. Moreover, enhancement of Cx43 function may play an important role in the antiarrhythmic effect of CIH. On the other hand, the arrhythmogenic effect of CIH seems to be associated with an increase in the plasma norepinephrine level, mostly in protocols mimicking sleeping apnea.

**Figure 3:**
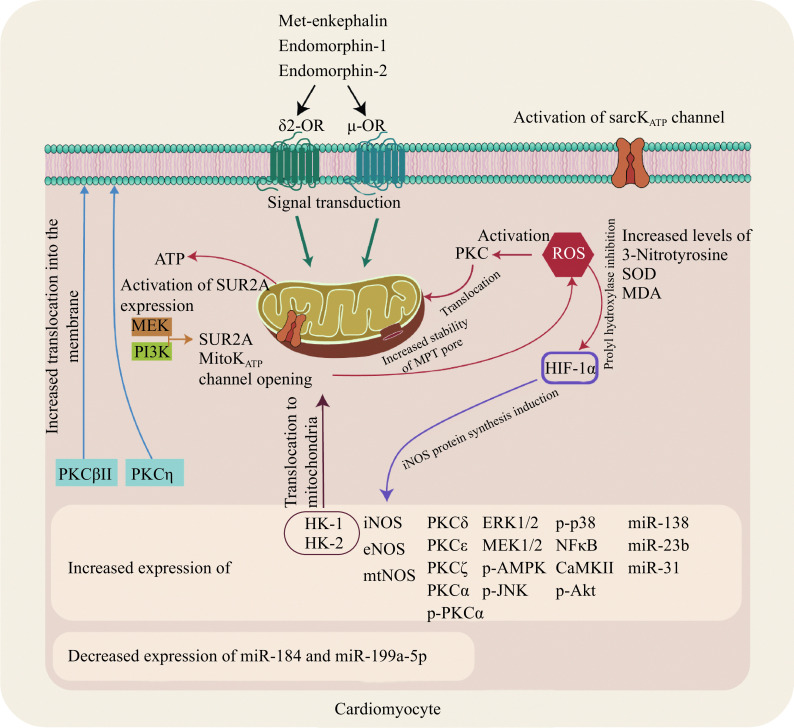
The effect of chronic hypoxia on the cellular and molecular mechanisms.

It was further demonstrated that CH provided the infarct-limiting effect and the vasoprotective effect in cardiac I/R as well as improved the recovery of cardiac contractility. At the humoral level, the infarct-reducing effect of CH may be mediated by catecholamines, cholinergic and endogenous opioids, ANP, erythropoietin, endothelin-1, adrenomedullin, and related membrane receptors. At the molecular level, the appropriate amount of ROS may serve as signaling molecules stimulating the cardioprotective pathways of CIH-induced cardiac tolerance to I/R. Particularly, the activation of iNOS, PKCε, PKCδ, CaMKⅡ, ERK1/2, MEK1/2, PI3K, Akt, p38, JNK, PKG, and AMPK may be involved in the cardioprotective mechanism. Although HIF-1α plays a key role, other inflammatory pathways may also be involved. It is also possible that miRNAs may participate in CH-induced cardioprotection. It was hypothesized that mitochondria could be the end-effector of CH-induced cardioprotection, because they play an important role in an enhancement of cardiac tolerance to I/R *via* mitoK_ATP_, and perhaps the mitochondrial BK_Ca_ channel activation is associated with the prevention of the MPT pore opening (***Fig. 3***).

The main complications of CH are pulmonary hypertension and subsequent RV hypertrophy. However, PH and RV hypertrophy can be reversible. CIH can improve clinical manifestation of CAD possibly by increasing the collateral blood flow in the heart. The cardioprotective effect of CH is developed slowly requiring 21 days for the development of cardiac tolerance to I/R. In addition, CH results in the formation of pulmonary hypertension. Therefore, it is unlikely that CH will find widespread use in clinical practice. CH has an important advantage over the ischemic preconditioning in that it induces a long-term increase in the heart's tolerance to I/R. Therefore, future studies of the molecular mechanisms of CH may open up prospects for the creation of new drugs for increasing cardiac tolerance to I/R. In our opinion, pharmacological mimicking of the cardioprotective effect of CH is much more promising than CH itself.
